# Thoracoscopic excision of mediastinal cysts in children

**DOI:** 10.4103/0972-9941.38905

**Published:** 2007

**Authors:** Prashant Jain, Beejal Sanghvi, Hemanshi Shah, S V Parelkar, S S Borwankar

**Affiliations:** Department of Pediatric Surgery, KEM Hospital Mumbai, India

**Keywords:** Bronchogenic cyst, mediastinal cyst, thoracoscopy

## Abstract

**Aim:**

Thoracoscopy offers great advantages when compared with open surgery in terms of postoperative pain and pulmonary complications. Considering the benign nature of most of the mediastinal cysts, thoracoscopy is safe and feasible with minimal morbidity. The purpose of this article is to review our experience with four cases of mediastinal cysts resected successfully within a period of one year by thoracoscopy.

**Materials and Methods:**

The cases of mediastinal cysts operated by thoracoscopic excision in K.E.M. Hospital, Mumbai from November 2005 to December 2006 were reviewed. The age varied from six months to 10 years. The patients presented with respiratory distress or recurrent lower respiratory tract infection. All patients underwent Chest X-ray and CT scan thorax to delineate the location of the cyst and its relationship with adjacent vital structures. Two patients had anterior and two had posterior mediastinal cyst. The ports were placed depending on the location of the cyst on the CT scan, following the principles of triangularization. The cysts were excised mainly by blunt dissection.

**Results:**

All the patients were successfully managed by thoracoscopic surgery. None of them had intraoperative complications. Dissection in patient with history of recurrent respiratory tract infection was difficult because of adhesions. Intercostal drain was removed within 48hrs and the patients were discharged on the fourth postoperative day.

**Conclusions:**

Thoracoscopy in mediastinal cysts is a safe and effective procedure with low morbidity and a shorter hospital stay.

## INTRODUCTION

Thoracoscopy offers great advantages when compared with open surgery in terms of postoperative pain and pulmonary complications. Thoracoscopy is now being increasingly utilized for various pediatric procedures like patent ductus arteriosus ligation, congenital diaphragmatic hernia repair, tracheo-esophageal fistula repair, lung resection etc. Considering the benign nature of most of the mediastinal cysts, thoracoscopic excision is a safe and feasible procedure with minimal morbidity and therefore finds its application even in asymptomatic patients.

Video assisted thoracoscopic surgery permits good exposure of the entire thoracic cavity including the mediastinum and better evaluation of the anatomic relationship. It provides adequate access and space for almost all maneuvers of dissection. Also, the surgical trauma is minimized.

## MATERIALS AND METHODS

The cases of mediastinal cysts operated by thoracoscopic excision in K.E.M. Hospital from November 2005 to December 2006 were reviewed. The age varied from six months to 10 years. The patients presented with complaints of respiratory distress or recurrent lower respiratory tract infection. All patients underwent Chest X-ray and CT scan thorax to delineate the location of the cyst and its association with adjacent vital structures. Magnetic resonance imaging was performed in one patient to rule out intraspinal communication. Two patients had anterior and two had posterior mediastinal cyst.

Thoracoscopy was done using conventional endotracheal ventilation. Patient was given lateral decubitus position with padding under opposite hemithorax. The trocars were placed depending on the location of the cyst on the CT scan, following the principle of triangularization. Three 5 mm trocars were used, one for thoracoscope and others for grasper/scissors/hook/aspiration needle. Insufflation of 5 mm Hg was used to create the pneumothorax and to maintain good field of vision. Cyst was first evaluated for its site and relationship with the adjacent structures. The pleura over the cyst was incised and dissected. To make the manipulation easy, cyst was initially aspirated and decompressed. Complete excision of the cyst was done mainly by blunt dissection and traction. Chest tube drain was inserted before the trocars were removed.

## CASE HISTORY

### Case 1:

A five-year-old male child was investigated for persistent breathlessness and cough of two years duration. Patient received treatment for open pulmonary tuberculosis one year back. He also had evidence of rickets with rachitic rosary. Skiagram chest showed persistent haziness in the right upper hemithorax. CT scan [Figure 1A] revealed a well-defined posterior mediastinal cyst separate from the pericardium and bronchi. Thoracoscopy showed a well-defined cyst in the posterior mediastinum [Figure 1B], which could be removed completely. Histopathology showed cyst wall lined by ciliated columnar epithelium suggestive of bronchogenic cyst.

**Figure 1A:**
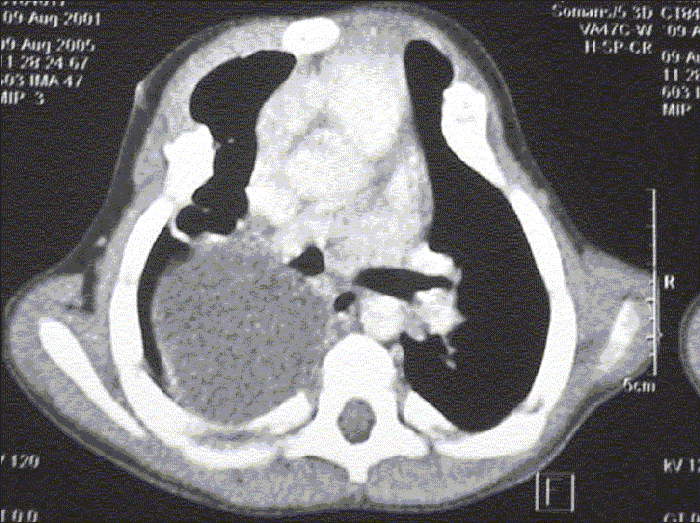
CT scan chest showing well defined posterior mediastinal cyst

**Figure 1B:**
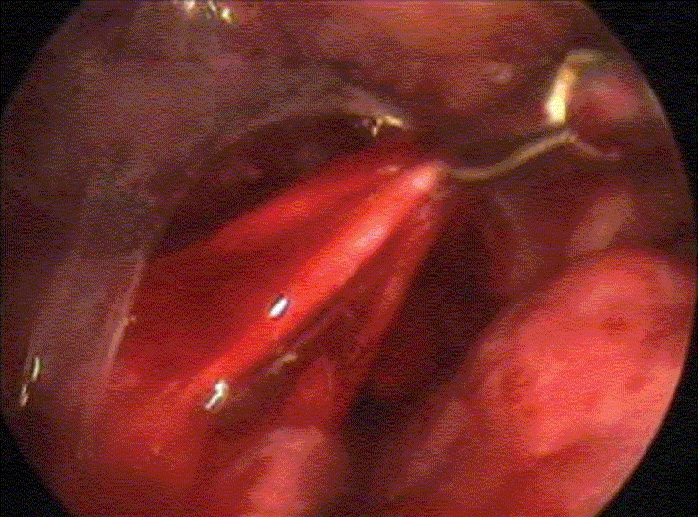
Dissection of posterior mediastinal cyst after incising the overlying mediastinal pleura

### Case 2:

**Figure 2:**
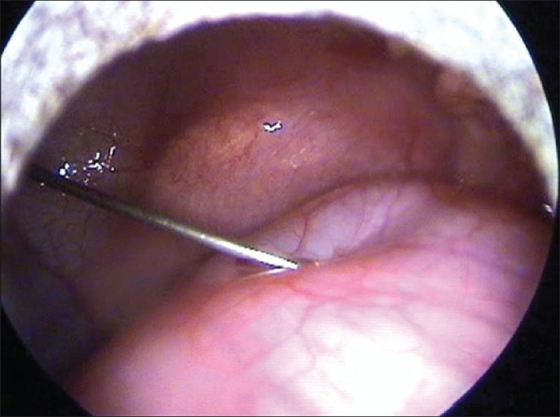
Aspiration of the cyst

A six-month-old male child presented with breathlessness since seven days. Patient had history of three episodes of lower respiratory tract infection. Skiagram chest revealed a right mediastinal mass with mediastinal shift to the opposite side. CT scan showed a posterior mediastinal cyst with coronal clefting of the seventh thoracic vertebra suggesting a neuroenteric cyst. MRI spine revealed no intraspinal communication. Cyst was initially aspirated [Figure 2]. It was densely adherent to the spine but there was no intraspinal communication. Histopathology showed cyst wall lined by columnar epithelium with gastric mucosa at places suggestive of enteric duplication cyst.

### Case 3:

**Figure 3:**
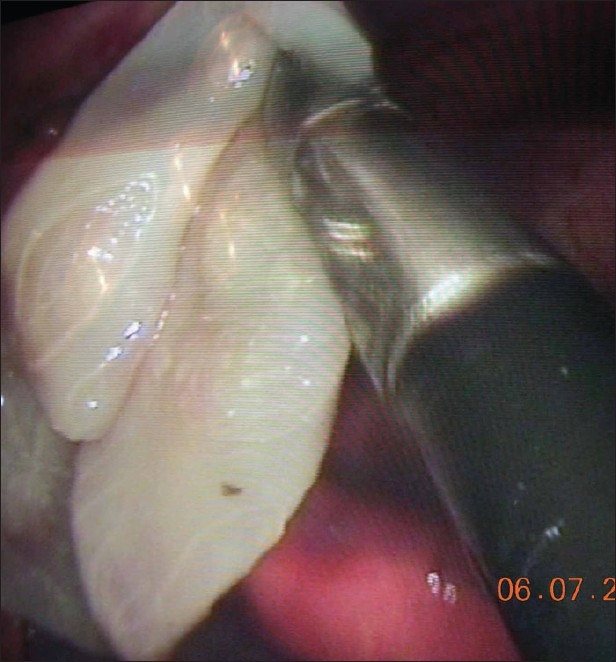
Thoracoscopic enucleation of hydatid cyst

A ten-year-old male child presented with recurrent cough. CT scan showed a well-defined cyst in the antero-superior mediastinum suggestive of a pericardial or a thymic cyst. On thoracoscopy, the cyst wall resembled an egg membrane with straw colored fluid within it, diagnostic of a hydatid cyst [Figure 3]. To prevent the development of secondary hydatid cysts because of spillage of hydatid fluid, povidone-iodine was used as a scolicidal agent. Whole of the cyst was removed en-sac. The patient was started on albendazole.

### Case 4:

A six-year-old male child presented with complaints of fever and chest pain since one week. X-ray and CT scan thorax were suggestive of a cystic lesion in the antero-superior mediastinum. The cyst was separate from the pericardium and was in close relation to the great vessels. On thoracoscopy, there was a large thick walled mediastinal cyst separate from the pericardium, closely adherent to the thymus. Sero-hemorrhagic fluid was aspirated and the cyst was excised. Histopathology was suggestive of a thymic cyst.

## RESULTS

All the patients were successfully managed by thoracoscopic surgery. None had intra-operative complications. In all patients cyst could be easily dissected from the pericardium, bronchi and major vessels. Size of the cyst varied from 4–6 cms. Dissection in one patient who had history of recurrent respiratory tract infection was difficult. The duration of surgery varied from 80 to 120 mints. Intercostal drain was removed within 48hrs and patients were discharged on the fourth postoperative day. All the patients are asymptomatic over a follow-up period of two months to 12 months.

## DISCUSSION

Thoracoscopy in children was first reported by Rodgers and Talbert in 1976.[1] Bronchogenic and other types of foregut cysts comprise 10–18% of all the mediastinal masses identified in infants and children and 20–32% of all mediastinal masses when all age groups are included. Symptoms are usually caused by compression of intrathoracic structures, with respiratory complaints predominating.[2]

CT scan is considered essential and important for displaying morphology, density and extent of mediastinal cysts. Also it is helpful in delineating any communication with esophagus or bronchus. Although CT scan could not confirm the diagnosis in one of our four cases (case 3), it often accurately defines the relationship of the lesion with the adjacent structures. In cases where a neurenteric cyst is suspected because of associated vertebral anomaly, a careful examination of the spinal extension of the lesion should be done using MRI and the spinal component should usually be approached first.[2]

Percutaneous or transbronchial aspiration, injection of sclerosing agents and excision *via* mediastinoscopy have also been reported as treatment modalities of these cysts. However, the recurrence rates with these methods are much higher than reported following surgical excision.[3]

Thoracoscopy has an expanding role in the treatment of Foregut Duplication cysts. The surgical principle of complete resection is used as followed in open surgery, which helped in reducing the recurrence.

Michel *et al,*[4] reported a series of 21 children with mediastinal cysts of which 18 were successfully treated by thoracoscopy, rest of them required thoracotomy because of difficult dissection. In only one of our patients with enteric duplication cyst, there were dense adhesions but complete excision was possible by blunt dissection. None of the patients had intraoperative complications. Thoracoscopy permitted good exposure of the entire mediastinum, adequate space for all maneuvers of dissection, easy access to various areas, better evaluation of the anatomic relationship and possibly, reduction of the surgical trauma. Bratu I *et al*,[2] with their experience of thoracoscopic excision in 11 patients stated that magnification provided by thoracoscopy may be advantageous for the complete meticulous excision of foregut duplication cysts in different locations. Creating artificial pneumothorax collapses the lung and gives good exposure.[5] Aspiration of the cyst improves the vision and facilitates the dissection.[2] The surgeon can convert to thoracotomy if the dissection is difficult.

If the cyst shares a common wall with the trachea or esophagus, this portion of the cyst wall is to be left behind and the mucosa is stripped or destroyed by electrocautrey.[6] The absence of pleural adhesions makes it possible to create large window to dissect the cyst.

Adhesions, large cysts and subcarinal cyst are associated with high conversion rates. Compressive cysts with lung distension and mediastinal shift are considered contraindication for thoracoscopy.[3] Dissection is more difficult in cases of infection, very large cysts and if the cyst is located under the carina. The absence of pleural adhesions makes it possible to create large pleural window.[4] In our cases cyst were of size 4–6 cms and only one of them was adherent to pleura. Besides the other obvious advantages, thoracoscopic resection reduces hospital stay and so it is also cost effective.[7]

## CONCLUSION

To conclude, thoracoscopy is an effective diagnostic and/or therapeutic method that can and should be included in the operational routine of thoracic surgery for the management of mediastinal cysts. Thoracoscopy is safe with low morbidity and shorter hospital stays and thus reducing the financial burden.
